# Temperature and aggression revisited: Evidence from 1 million amateur football matches

**DOI:** 10.1093/pnasnexus/pgag202

**Published:** 2026-06-12

**Authors:** Sascha Riaz, Maurice Baudet von Gersdorff, Heike Klüver

**Affiliations:** Department of Political and Social Sciences, European University Institute, San Domenico di Fiesole 50014, Tuscany, Italy; Department of Social Sciences, Humboldt-Universität zu Berlin, Berlin 10099, Germany; Department of Social Sciences, Humboldt-Universität zu Berlin, Berlin 10099, Germany

**Keywords:** temperature, aggression, conflict, climate, football

## Abstract

A large body of research documents a positive relationship between temperature and human aggression, underpinning projections of increased conflict in a warming climate. However, most prior evidence relies on laboratory experiments with convenience samples or on crime records, both of which are limited in their generalizability to everyday social interactions. To isolate the effect of temperature in a standardized, real-world social environment, we analyze ∼1 million amateur football matches played across Germany and study aggression among more than one million amateur players. Comparing matches played at the same venue within the same league and season, and adjusting for referee and team differences, we find a precisely estimated inverted-U relationship between temperature and player aggression: disciplinary cards (issued by referees for fouls and misconduct) increase with temperature up to ∼13 °C but decline thereafter. Matches played under extreme heat show a 15% reduction in disciplinary actions relative to the sample mean. Our results contribute to the temperature–aggression literature: in line with affect-based accounts of aggression, moderate warmth may increase irritability and arousal; however, to the extent that aggressive behavior requires physical engagement, it may decline at extreme temperatures.

A large literature links high temperatures to human aggression and conflict (1, 2). Extreme heat has been associated with assaults (3), domestic violence (4), and broader social instability (5), informing projections that anthropogenic climate change may increase violent conflict (1, 2). Yet, much of the underlying evidence comes from settings with limited generalizability: (i) observational crime data, where heat alters the routine activities of perpetrators and victims (6), offenders are a highly selected subset of the population, and crime depends on victim behavior that itself varies with temperature; (ii) laboratory experiments with convenience samples that abstract away from the physical and social constraints of everyday interactions (7); and (iii) professional sports settings, where athletes represent a highly selected population (8). To address these limitations and study the temperature–aggression link in standardized, real-world encounters, we turn to amateur football (soccer).

Amateur team sports provide a controlled yet naturalistic setting to examine the temperature–aggression link among ordinary citizens at scale. Throughout, our focus is on player aggression on the pitch, not on crowd or spectator behavior. We analyze ∼1 million football matches played across Germany between July 2022 and September 2025, involving more than one million unique players across different age groups, for both men and women (though predominantly male; 8.4% of matches come from women’s leagues). Our sample covers the amateur tiers of the German football league pyramid administered by the German Football Association (DFB), excluding the three professional divisions (Bundesliga, 2. Bundesliga, 3. Liga) and ranging from the semiprofessional Regionalliga (tier 4) down to recreational district- and county-level competitions, open to any player registered with a DFB-affiliated club. The setting offers several advantages: matches are scheduled weeks in advance, making fixture timing independent of weather; rules of play are standardized; physical activity cannot be avoided; and players generally cannot exit the match. We use disciplinary cards as a proxy for player aggression. Yellow cards are formal warnings issued for moderate fouls or unsporting conduct; red cards entail immediate ejection and are reserved for serious offenses such as violent conduct, reckless play that risks injuring an opponent, or denying a clear goal-scoring opportunity.^a^ While cards do not exclusively reflect hostile intent (some sanction tactical fouls or handballs), they capture observable rule violations that often involve physically aggressive contact and confrontational interactions.

For our empirical analysis, we link each match to the maximum air temperature in a 3-h window from kickoff, measured at the nearest of ∼450 weather stations (median distance 10.3 km). We estimate a quadratic specification with fixed effects for season × league × postal code, referee, and team, so that identification comes from temperature variation within the same venue, league, and season, not differences across regions or teams (see Materials and methods for details).

Several theoretical frameworks offer accounts of the temperature–aggression link. The general aggression model attributes it to heat-induced increases in hostile affect and physiological arousal (9). Routine activity theory traces it instead to weather-driven changes in outdoor social contact, with pleasant temperatures pushing people outdoors and increasing the rate of conflict-prone encounters (10). The negative affect escape model holds that ambient discomfort raises aggression up to a threshold, beyond which people seek to leave the situation rather than retaliate (11). Social escape and avoidance theory integrates these accounts, with extreme temperatures driving voluntary withdrawal from outdoor contact (12). A key feature of our setting is that the central mechanisms underlying the latter three frameworks (changes in social contact and the option to disengage) are closed by design: social contact is institutionally fixed at 22 players regardless of weather. Our setting thus allows us to study the temperature–aggression link with the contact and exit channels held closed.

## Results

**Figure 1:**
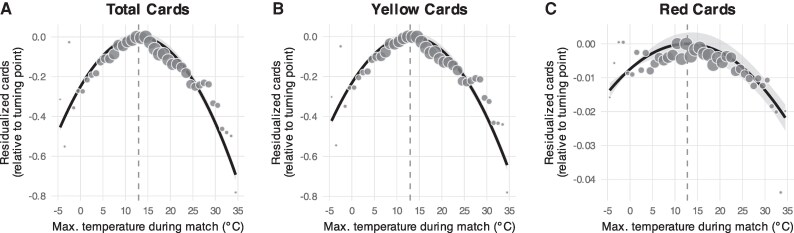
Relationship between match-day maximum temperature and disciplinary cards. Each panel shows binned means of residualized outcomes (dots, sized proportional to bin count) after partialing out season × league × postal code, referee, home team, and away team fixed effects. Solid curves are parametric quadratic fits; shaded bands indicate pointwise 95% CI. Vertical dashed lines mark the estimated turning point. A) Total cards (yellow + red + second yellow). B) Yellow cards only. C) Red cards only. N=997,586 matches; SE clustered at the postal code × day level.

Figure 1 presents binned scatterplots of residualized disciplinary cards against maximum match-day temperature (see Materials and methods for details). The relationship is clearly nonlinear. Across a series of specifications, we find a precisely estimated inverted-U relationship: cards increase with temperature up to ∼13 °C (both linear and quadratic terms P<0.001), then decline. This pattern is visible in the binned Fig. 1 and grows stronger, not weaker, as the comparisons become more granular and account for more confounders. We find similar results for yellow cards and red cards, though the results are more pronounced for yellow cards (Fig. 1B and C).

Table 1 reports results from a binary specification in which matches are classified as extreme temperature if the maximum temperature exceeds 32 °C (N=2,444; the top 0.2% of the sample distribution). Based on the preferred specification (column 4), extreme temperature reduces total cards by roughly 15% relative to the sample mean (P<0.001). The results also hold when adding calendar month to the fixed effects, so that identification relies exclusively on within-month temperature shocks (Table 1, column 5).^b^

**Table 1.** Effect of extreme heat on disciplinary cards.

	(1)	(2)	(3)	(4)	(5)
Extreme temperature (≥32 °C)	−0.2423***	−0.3252***	−0.4297***	−0.4636***	−0.3816***
	(0.0494)	(0.0472)	(0.0475)	(0.0497)	(0.0738)
*Fixed effects*					
Season × League	✓	✓	✓	✓	✓
× Postal code				✓	✓
× Month					✓
Referee		✓	✓	✓	✓
Home team			✓	✓	✓
Away team			✓	✓	✓
Observations	997,584	986,784	985,229	980,006	792,121
Dep. var. mean	3.05	3.05	3.05	3.05	3.05

The outcome is total cards per match (yellow + red + second yellow, both teams). Extreme temperature is a binary indicator for matches where the maximum temperature during the match exceeds 32 °C. SE clustered at the postal code×day level in parentheses. ***P < 0.001.

The inverted-U relationship is robust to (i) a cubic specification, (ii) controlling for weather station elevation, (iii) using apparent temperature (heat index) instead of dry-bulb temperature,^c^ (iv) restricting to adult matches (79% of observations), (v) clustering SE at the postal code level, (vi) controlling for precipitation during the match, and (vii) adding day-of-week and kickoff hour as controls. The pattern also holds when stratifying by gender: although the overall level of cards is much lower in women’s football (mean of 0.75 vs. 3.26 cards in male matches), the quadratic relationship is statistically significant in both subsamples, with estimated turning points of 12.9 °C in male leagues and 14.7 °C in women’s leagues.

## Discussion

We document an inverted-U relationship between temperature and disciplinary cards in amateur football. This finding implies a scope condition for the temperature–aggression literature: in line with affect-based accounts of aggression, moderate warmth may increase irritability and arousal; however, to the extent that aggressive encounters require physical engagement, they may decline at extreme temperatures. This implies that a negative relationship between temperature and conflict can arise even when the exit channel emphasized by the Negative Affect Escape (11) and Social Escape and Avoidance (12) frameworks is closed by design. Whether the same dynamic extends to different settings (criminal violence, political conflict) remains an open question. Another direction for future research is to examine temperature *anomalies*, deviations between realized and expected temperatures for a given location and date, rather than absolute temperatures.

While we do not have direct evidence on the mechanism, we view a reduction in physical engagement as the most likely driver of our results. Card-worthy fouls often result from players operating at the limits of their physical capacity: mistimed tackles, late challenges in which a defender arrives after the ball, contested headers, and full-effort duels. When players downshift effort under thermal stress, the rate of such high-intensity interactions falls, and with it the rate at which they tip into fouls. This is consistent with two of our empirical findings. First, the estimated 13 °C turning point falls within the temperature range shown by prior research to maximize prolonged aerobic exercise capacity (13). Second, the decline at high temperatures is stronger for yellow than red cards: yellow cards often sanction byproducts of high-intensity contact, whereas red cards more often reflect offenses unrelated to physical effort, such as abusive language toward officials or denial of a clear goal-scoring opportunity.

A possible alternative mechanism is that referee decision-making varies with temperature. In light of prior research, we view this as unlikely: previous research (14) finds no effect of thermal condition on the decision-making accuracy of soccer referees in a match-simulation protocol. Moreover, the physical engagement we view as the most likely driver of our results is generally more demanding for players than for referees, whose role depends more heavily on cognition; meta-analytic evidence places the cognitive-performance optimum around 21–27 °C (15), well above the 13 °C turning point we estimate.

## Materials and methods

### Football match-level data

We analyze the universe of amateur football matches recorded on *fussball.de*, the official platform of the German Football Association (DFB), played between July 2022 and September 2025. The DFB and its 21 regional associations administer the league pyramid. The amateur tiers we analyze span the semiprofessional Regionalliga (tier 4), Oberliga (tier 5), state-level divisions, and recreational competitions (Bezirksliga, Kreisliga, and Kreisklasse) and include men’s, women’s, and youth leagues. The final sample comprises 997,586 matches across 5,638 postal codes, officiated by 48,305 unique referees. The outcome variable is total disciplinary cards per match (yellow, red, and second-yellow cards summed across both teams; mean=3.05, SD=2.53). Coverage of Bavaria is limited to upper-tier leagues (2,883 matches, 0.3% of all observations); Bavarian data are hosted on a different website.

### Weather data

Hourly air temperature observations are obtained from ∼450 stations of the German Weather Service (Deutscher Wetterdienst, DWD). For each match, we identify the nearest weather station to the venue’s postal code centroid (median distance: 10.3 km) and compute the maximum temperature during a 3-h window starting at kickoff. Maximum match-day temperature ranges from −8.0 to 38.3 °C (mean =15.5 °C, SD =6.5 °C). As a robustness check, we also use apparent temperature (*gefühlte Temperatur*).

### Model specification

We estimate:


(1) $${\text{Cards}}_{i}=\beta_{1}{\text{Temp}}_{i}+\beta_{2}{\text{Temp}}_{i}^{2}+\alpha +\varepsilon_{i},$$

where α denotes fixed effects for season × league × postal code (79,892 cells, where league denotes the division × age bracket × gender combination), referee (48,305), home team (43,908), and away team (44,330), absorbing a total of 216,435 parameters. SE are clustered at the postal code×day level (570,977 clusters). We also estimate a binary specification replacing the quadratic temperature terms with an indicator for extreme temperature (≥32 °C).

### Binned figure

Figure 1 plots binned means of residualized outcomes against maximum match-day temperature. For each outcome, we first partial out all variation attributable to the full set of fixed effects (season × league × postal code, referee, home team, and away team). The residuals are then grouped into 1 °C temperature bins, and the within-bin mean is plotted as a dot sized proportional to the number of matches in that bin.

## Data Availability

Replication data and code are available at https://github.com/saschariaz/cards_temp.

## Notes

a A player who receives two yellow cards in the same match is also ejected.

b The 32 °C cutoff corresponds to what the German Weather Service (DWD) defines as *starke Wärmebelastung* (strong heat stress), though on the basis of apparent temperature (*gefühlte Temperatur*).

c Apparent temperature is almost perfectly correlated with dry-bulb temperature in our sample (Pearson r>0.99) and yields an inverted-U with an estimated turning point at 11.8 °C, very similar to our main results.
